# Contrast-Enhanced Ultrasound to Assess Kidney Quality During *Ex Situ* Normothermic Machine Perfusion

**DOI:** 10.3389/ti.2025.14268

**Published:** 2025-04-02

**Authors:** Samuel J. Tingle, Chloe Connelly, Emily K. Glover, Ben Stenberg, Andrew McNeill, Georgios Kourounis, Beth G. Gibson, Balaji Mahendran, Lucy Bates, Madison N. Cooper, Rhys R. Pook, Samantha Lee, Marnie L. Brown, Rodrigo Figueiredo, Kevin J. Marchbank, Simi Ali, Neil S. Sheerin, Colin H. Wilson, Emily R. Thompson

**Affiliations:** ^1^ Translational and Clinical Research Institute, Newcastle University, Newcastle upon Tyne, United Kingdom; ^2^ National Institute for Health and Care Research Blood and Transplant Research Unit, Newcastle University and Cambridge University, Newcastle upon Tyne, United Kingdom; ^3^ Institute of Transplantation, Freeman Hospital, Newcastle upon Tyne, United Kingdom; ^4^ Renal Services, Newcastle Upon Tyne Hospitals NHS Foundation Trust, Newcastle upon Tyne, United Kingdom; ^5^ Department of Radiology, Freeman Hospital, Newcastle upon Tyne, United Kingdom

**Keywords:** kidney transplantation, machine perfusion, contrast enhanced ultrasound, viability assessment, assessment and reconditioning, time intensity curve, ischaemia reperfusion, R package

## Abstract

Normothermic machine perfusion (NMP) provides opportunity for viability assessment of donated kidneys. Diminished microvascular perfusion, despite adequate total blood flow, is a key pathophysiology in ischaemia-mediated acute kidney injury. Contrast-enhanced ultrasound (CEUS) could allow objective assessment of microvascular perfusion during renal NMP. Blood-based NMP was performed on porcine kidneys (circulatory death model) and human kidneys declined for transplant (preclinical). CEUS was performed with a contrast bolus into the NMP circuit arterial limb. Microvascular perfusion quality was quantified and z-score normalisation allowed combination of metrics and regions into an overall “CEUS-score.” In porcine kidneys, inferior microvascular perfusion of cortex and medulla correlated with increased urinary NGAL (Neutrophil gelatinase-associated lipocalin) and histological DNA-fragmentation (a hallmark of apoptosis). In human kidneys, CEUS-score at 2 h was correlated with histological DNA-fragmentation (r = −0.937; P = 0.019) and predicted urinary NGAL at 24 h of NMP (r = −0.925; P = 0.024). Total renal flow was not correlated with these outcomes. An open-source web application (stingle.shinyapps.io/Time_intensity_analysis) and R package (“tican”) were developed for quantitative time-intensity curve analysis. CEUS allows objective point-of-care microvascular perfusion assessment during NMP. As 2-hour CEUS-score predicts NGAL at 24 h, CEUS warrants future clinical investigation as a potential tool to assess kidney quality in assessment and reconditioning centres.

## Graphical Abstract

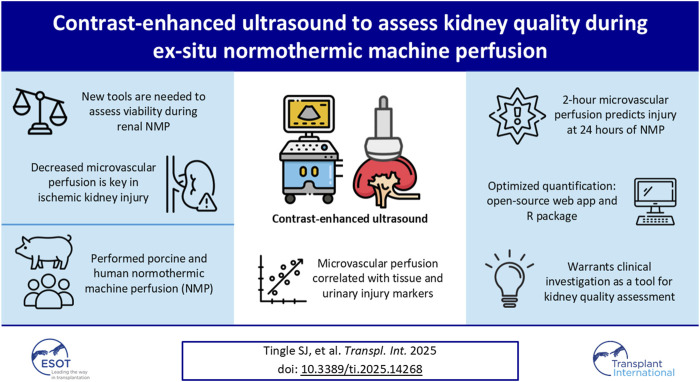

## Introduction

International disparities between waiting list demands and availability of suitable donor organs drive new approaches to increase the donor pool. As such, there is an increased use of marginal organs from extended criteria donors. Technologies that can improve selection, allocation and utilisation of organs with confidence are vital to ensure we meet the demands of the waiting list. *Ex situ* normothermic machine perfusion (NMP) of isolated organs offers an optimal platform for viability assessment of marginal kidneys prior to transplantation.

The majority of the validated tools used to assess organ quality during machine perfusion have been based on biochemical read-outs, blood flow rate or urine output [1]. In renal transplantation the “Quality Assessment Score” combines urine output by 1 h, with renal blood flow and visual assessment of global perfusion at 1 h [2, 3]. However, this did not correlate with kidney transplant outcomes in a large randomised controlled trial, driving the need for improved assessment modalities [4].

Poor renal microvascular perfusion, despite adequate total renal blood flow, is increasingly seen as one of the hallmarks of renal ischaemia reperfusion injury in the setting of acute kidney injury (AKI) [4, 5]. Contrast-enhanced ultrasound (CEUS) is an imaging technique commonly used in clinical practice, including post-liver and kidney transplantation, to assess microvascular perfusion [6]. CEUS utilises the infusion of microbubbles of sulphur hexafluoride in a phospholipid shell into the circulation. These bubbles are small enough to reach the capillary bed but not small enough to pass out into the interstitium therefore giving a global picture of tissue perfusion. This avoids the radiation and nephrotoxicity of alternative imaging/contrast techniques [6].

In our group, we have experience of using contrast enhanced ultrasound (CEUS) during hypothermic machine perfusion of kidneys and we have developed this technology to apply it to normothermic machine perfusion of both livers and kidneys [7, 8]. We have also previously demonstrated that CEUS was a valuable tool in the assessment of a cellular therapy delivered during machine perfusion [9]. However, no study to date has assessed the validity of the use of CEUS as a viability tool during renal NMP. This study aimed to develop CEUS as a potential viability assessment tool for human kidneys undergoing NMP.

## Materials and Methods

### Porcine Circulatory Death Model

All animals were euthanised by overdose of anaesthetic according to schedule 1 of the United Kingdom Animals (Scientific Procedures) Act 1986. Use of animals and collection of kidneys for these studies was approved after a full ethical review by Newcastle Universities Animal Welfare and Ethical Review Board and ongoing review via study plan approval (AWERB number 854, study plan 38). Porcine kidneys were retrieved from 60 kg 16-week-old white landrace pigs. These were sedated using intramuscular injection of approximately 5 mL Tiletamine and Zolazepam (Zoletil™, Virbac). Pigs were then euthanised using ear vein injection of 25 mL (Euthatal™, Dopharma Research B.V.). Approximately 500 mL of blood was collected using standard clinical blood bags depleted of CDPA-1 and filled with 10,000 IU heparin and 50 mL saline (Fresenius Kabi). Kidneys were retrieved and kept in the body cavity until 25 min after confirmation of death, then flushed with 1 L of 4°C University of Wisconsin solution with 25,000 IU sodium heparin (Panpharma). This standardised the warm ischaemic time (WIT) to 25 min. Kidneys were kept on ice for 16 h of cold ischaemic time (CIT) prior to initiation of machine perfusion. All porcine kidneys reported here came from different pigs.

We chose a WIT of 25 min followed by CIT of 16 h following a previous series of optimisation experiments which explored a range of ischaemic times (data not published). These ischaemic times provide sufficient ischaemia to mimic the injury seen during NMP of extended criteria human kidneys. This is the same protocol used in our previous studies of therapeutic delivery during porcine NMP [10].

### Human Kidneys

Human kidneys retrieved for transplant but then deemed unsuitable were included. Ethical approval for accepting these kidneys was granted by the national research ethics commission in the United Kingdom, National Research Ethics System (15/SC/0161). We gained approvals for this project from the National Health Services Blood & Transplant’s (NHSBT) Research Innovation and Novel Technologies Advisory Group (RINTAG), who oversee the allocation of such research organs to authorized research groups. In all cases donor families provided generic consent to approved research projects.

### Machine Perfusion

NMP was performed using a customised Medtronic pediatric cardiopulmonary bypass system. The renal artery was cannulated, and oxygenated perfusate with red blood cells was perfused at 37°C (continuous flow), aiming for a mean arterial pressure of 75 mmHg. Porcine and human perfusions differed in terms of their duration (6 h and 24 h respectively), source of blood (autologous whole blood versus packed red cells) and perfusate constituents. The increased duration in human versus porcine experiments reflects the growing international research interest in prolonged kidney perfusion and the recent establishment of these extended NMP protocols in our own perfusion laboratories [11]. Full protocols and lists of perfusate constituents for the two protocols is given in Supplementary Tables S1, S2. Total renal blood flow was measured using the Medtronic flow sensor (TX50P flow transducer, Medtronic). Urine production rate was measured via a paediatric nasogastric tube tied into the ureter. Oxygen consumption was calculated from blood gas and flow data, as previously described [12].

### CEUS

Ultrasound was performed with an eL18-4 probe of the Philips EPIQ7 Ultrasound machine with QLab 8.1 software (Philips). The probe (with sterile cover) was placed directly onto the kidney and held stationary to capture a longitudinal view of the kidney.

For CEUS imaging, 1 mL sulphur hexafluoride contrast agent (SonoVue ^®^, Bracco) was reconstituted with 4 mL of perfusate in a syringe. The contrast agent was then administered via a three way tap to the arterial limb of the circuit as a rapid bolus. A detailed standard operating procedure for capturing these cine loop recordings is available in Supplementary Table S3. We analysed CEUS scans from 6 h of perfusion (end of perfusion) for porcine kidneys and 2 h of perfusion for human kidneys. Each scan represents a single recording following a single bolus injection of contrast (recording was continued for a minimum of 60 s after contrast was seen reaching the kidney).

The built-in Philips “Contrast” mode was used; this is optimised for capturing microbubble contrast whilst minimising signal from any human tissue. When using the settings detailed in our standard operating procedure (Supplementary Table S3) this resulted in zero or negligible background signal, eliminating the need for normalisation of peak intensity to baseline value.

### CEUS Quantification Analysis

**FIGURE 1:**
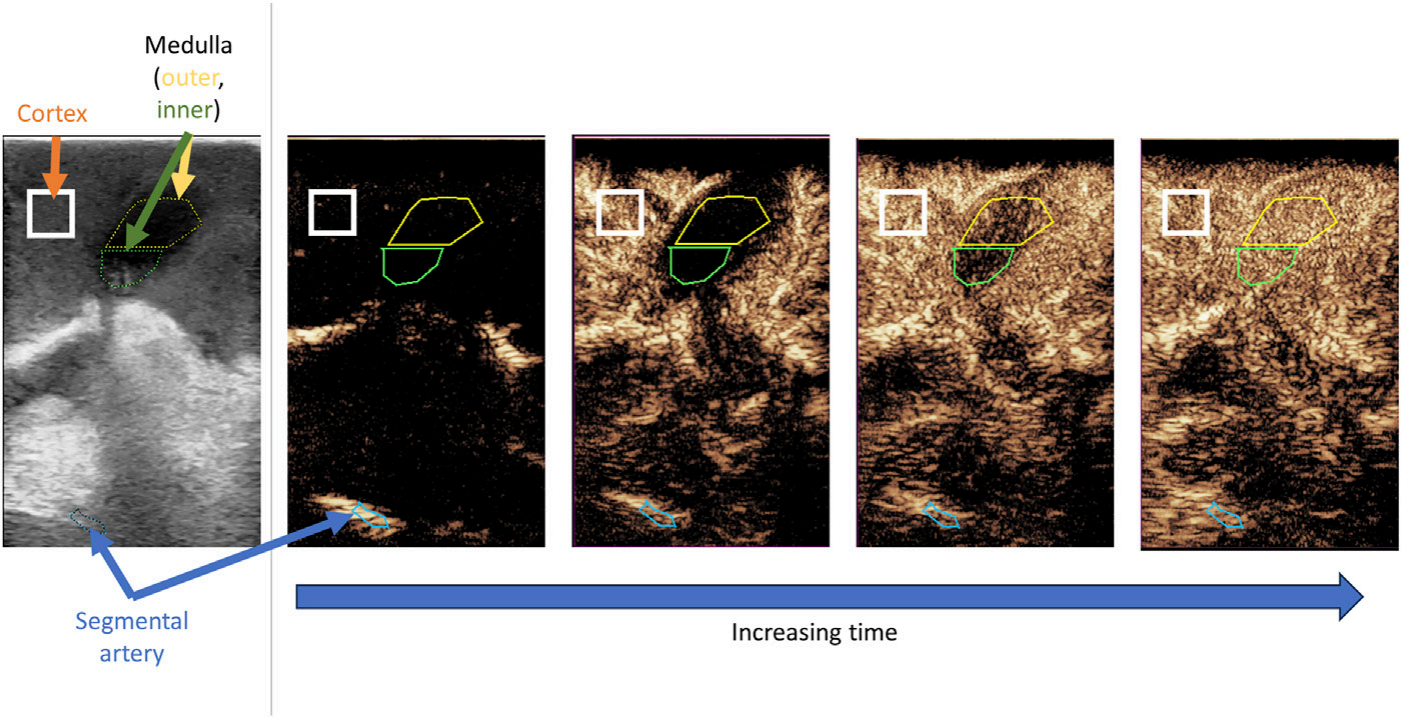
Representative contrast enhanced ultrasound loop with regions of interest selected. Standard B mode ultrasound image (left panel) is used to ensure cortex and medulla are in view. As time progresses there is sequential perfusion of segmental artery, cortex, outer medulla and inner medulla.

Raw CEUS DICOM files were imported into QLAB Advanced Quantification Software (Release 15.5 Philips) and analysed using the ROI QApp to get mean intensity for various regions of interest. A 5 × 5 mm square was used for the cortex. A freeform polygon was used to draw further regions of interest around the medulla. This was split equally into outer medulla (closest to the kidney surface) or inner medulla (closest to kidney hilum). Figure 1 displays drawing the regions of interest.

All cineloops were cut so that time zero was the frame that contrast was first seen (in segmental/interlobar arteries). All clips were cut to 30 s total length (selected as this is significantly longer than the time required for all regions to reach peak intensity). The ROI QApp then calculated the mean contrast intensity in the various regions of interest, for each frame of the ultrasound loop. This raw data (mean pixel intensity in decibels on every timestamped frame) for each region of interest was then exported for downstream analysis.

Analysis of CEUS data was performed in R (R Foundation for Statistical Computing, Vienna, Austria) [13]. A curve was plotted to the raw data, using a LOESS smoother. This was performed using the loess() base R function with loess.span set to 0.06 [13]. Data was extracted from this curve to calculate the peak intensity of contrast and time-to-peak intensity.

### Assays for Tissue DNA Fragmentation and Urinary NGAL

A TUNEL (TdT-mediated dUTP Nick-End Labelling) assay was performed on 4 µm FFPE sections. The DeadEnd™ Fluorometric TUNEL System (Promega) was carried out according to manufacturer’s instructions. Slides were mounted using VECTASHIELD mounting medium with DAPI (Vector labs). Cells with DNA fragmentation (as a hallmark of apoptosis) and DAPI-stained nuclei were counted using Fiji ImageJ software. Persons performing TUNEL assay and image analysis were blinded to CEUS data.

Neutrophil gelatinase-associated lipocalin (NGAL) concentration in urine was analysed by ELISA (DuoSet Cat: DY1757 for human, Abcam Cat: ab207924 for porcine). This was multiplied by urine production rate to get total nanograms of urinary NGAL per minute.

### Quantifying Red Cell Aggregates

Martius Scarlet Blue (MSB) staining was used to visualise erythrocytes, red cells, fibrin and collagen. Following dewaxing and rehydration, tissue was stained using a Martius Scarlet Blue Stain Kit (Atom Scientific) according to manufacturer’s instructions. LABKIT, a Fiji ImageJ plugin for segmentation of microscopy images was used to create a pixel classifier [14]. This enabled automatic segmentation of fibrin rich red cell aggregates (representative images in Supplementary Figure S1), which could be used to calculate the percentage of each image containing such aggregates.

### Statistical Analysis

To generate a single score for each region normalised values were required; z-scores for peak intensity and time-to-peak were therefore calculated [15]. The z-score for time-to-peak (TTP) could then be subtracted from the peak intensity (PI) z-score, such that the score for each region was calculated as follows:

$$Region\;CEUS\;score=\frac{\text{Sample}\;\text{PI}-\text{cohort}\;\text{mean}\;\text{PI}}{\text{cohort}\;\text{standard}\;\text{deviation}\;\text{PI}}-\frac{\text{Sample}\;\text{TTP}-\text{cohort}\;\text{mean}\;\text{TTP}}{\text{cohort}\;\text{standard}\;\text{deviation}\;\text{TTP}}$$

A table of the cohort average and standard deviation for peak and time-to-peak for each region, which were used to calculate these normalised z-scores, is given in Supplementary Table S4. As these are all normalised and on the z-score scale, scores for the three regions were added to generate an overall score for each kidney [15].

The correlation between CEUS metrics and NMP outcomes was assessed using the Pearson correlation coefficient. All statistical analyses were performed in R [13].

## Results

An annotated example CEUS cine loop can be viewed in the Supplementary Video. In total, 8 porcine kidneys and 5 human kidneys were included. Porcine kidneys came from 8 separate female donors. 3 kidneys were left and 5 were right.

### CEUS Is Associated With Urinary NGAL and Tissue Apoptosis in Porcine Kidneys

**FIGURE 2:**
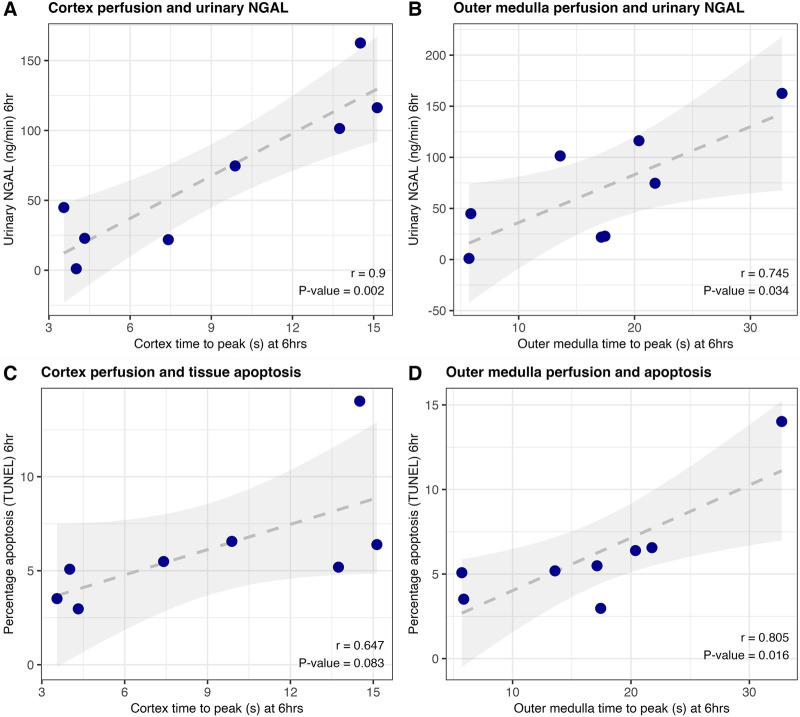
Correlation of CEUS results and urinary NGAL **(A, B)** or tissue DNA fragmentation (a hallmark of apoptosis) on TUNEL staining **(C, D)** at end of porcine machine perfusion (6 h); n = 8. Pearson correlation coefficient and associated p-value are displayed.

As shown in Figures 2A, B, perfusion of the cortex and medulla was correlated with urinary NGAL, an important predictor of kidney quality during NMP [16, 17]. Increasing time-to-peak (indicating worse microvascular perfusion) was associated with higher levels of damage marker NGAL (r = 0.90, P = 0.002 and r = 0.745, P = 0.034 for cortex and outer medulla respectively).

Similarly, improved quality of perfusion in these two regions was associated with a lower proportion of cells with DNA fragmentation (a hallmark of apoptosis) on histology (Figures 2C, D). Representative TUNEL images from kidneys with relatively poor versus good microvascular perfusion are shown in Supplementary Figure S2. There was no significant correlation between any CEUS metric and 6-hour urine flow rate.

### Negative Correlation Between Medullary Perfusion and Total Blood Flow Suggests Shunting

In the setting of AKI, shunting of blood through the kidney without parenchymal perfusion has been described as a key pathophysiological factor [4, 5]. We therefore correlated total blood flow through the kidney with parenchymal perfusion at an identical timepoint.

**FIGURE 3:**
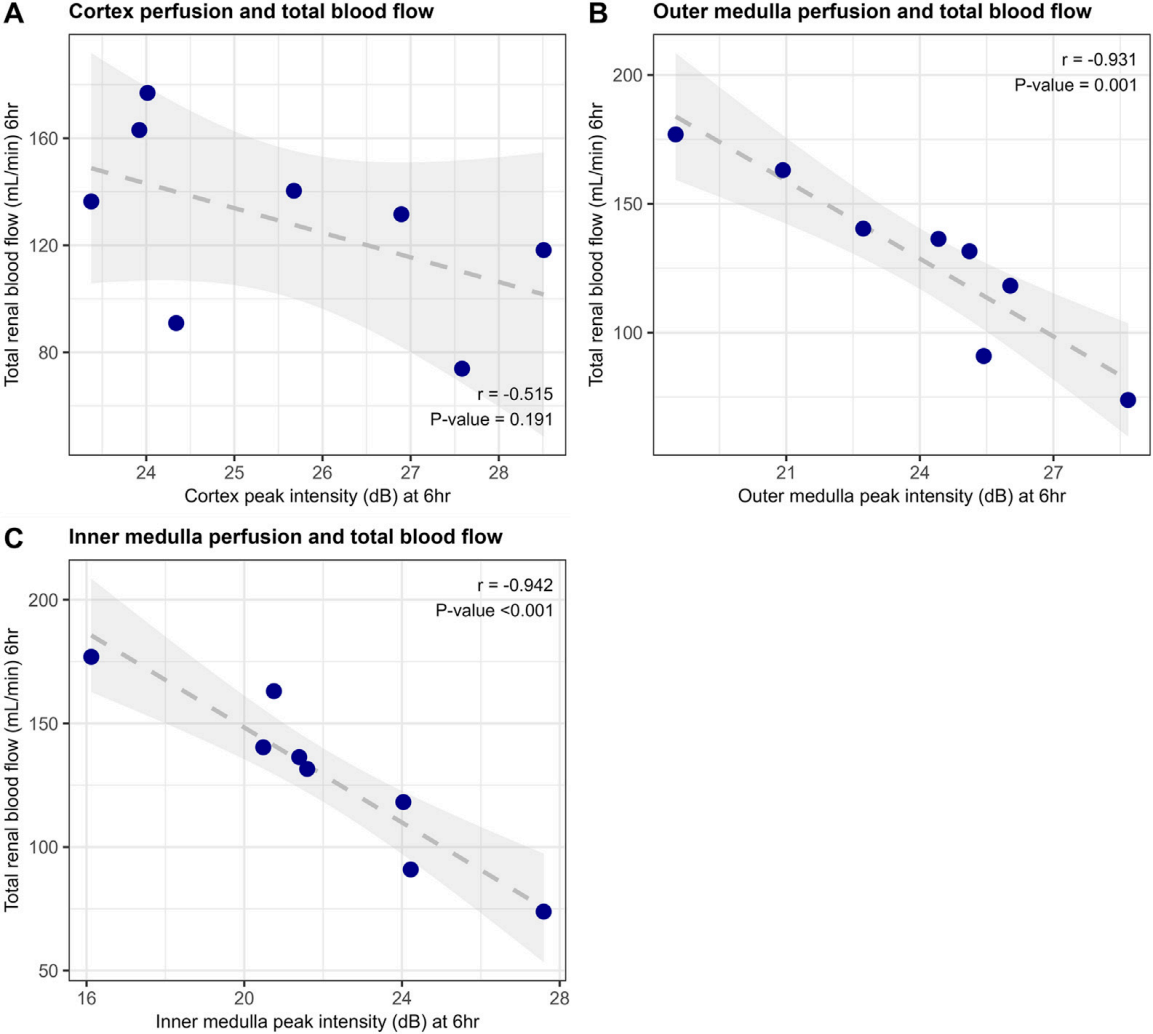
Correlation of end of machine perfusion (6 h) total renal blood flow and CEUS microvascular perfusion of cortex **(A)**, outer medulla **(B)** and inner medulla **(C)** in porcine kidneys (n = 8).

As shown in Figure 3A there was no significant correlation between cortex perfusion and total renal blood flow. However, there was a strong negative correlation between the quality of microvascular perfusion of the medulla and total renal blood flow (Figures 3B, C), indicating that more severe injury leads to shunting through low resistance vessels, with increased total flow but decreased parenchymal perfusion.

One potential contributing factor to the lack of microvascular perfusion is the presence of capillary obstruction by red cell aggregates, which have been reported during NMP previously [18, 19]. We found no correlation between burden of red blood cell microvascular occlusion and microvascular perfusion of the cortex or medulla at 6 h (Supplementary Figures S1, S3). Potentially indicating the role of shunting as opposed to occlusion.

### Validation of CEUS in Human Kidney Cohort

Human kidneys from five deceased donors were included. Donor demographics are provided in Table 1. Three kidneys were rejected due to extra-renal malignancy, one due to presence of glomerulosclerosis on biopsy and CIT, and one due to a significantly calcified aortic patch. None of the donors received normothermic regional perfusion.

**TABLE 1:** Cohort demographics, with one column per human donor.

Variable	Demographics for each kidney				
Cold ischaemia time (hours)	15.5	18	29	29.5	13
Donor age	42	72	73	73	79
Donor sex	F	F	M	M	F
Donor type	DBD	DCD	DBD	DBD	DCD
WIT (WLST to aortic cold flush)	N/A	104	N/A	N/A	29
Donor hypertension	No	Yes	No	Yes	No
Creatinine at retrieval (µmol/L)	166	50	77	76	55
Creatinine at admission (µmol/L)	98	68	83	80	48
Cause of death	HBD	HBD	HBD	ICH	ICH
Quality of cold perfusion (retrieval surgeon)	Good	Good	Good	Good	Good
UKDRI 2019^a^	1.09 (D2)	1.95 (D4)	2.08 (D4)	1.79 (D4)	2.11 (D4)
Quality assessment score^b^	3	3	4	3	2

DBD, donation following circulatory death; DCD, donation following brainstem death; HBD, hypoxic brain death; ICH, intracranial haemorrhage.

^a^ UK kidney donor risk index 2019 version; score and quartile given where D1 is the best quartile and D4 is the worst quartile [35].

^b^ Quality assessment score at 1 h as described in Hosgood et al. [2].

The association of CEUS metrics with both tissue DNA fragmentation and urinary NGAL was assessed. Mirroring the porcine results, improved microvascular perfusion was correlated with lower levels of tissue DNA fragmentation, and lower levels of the damage marker NGAL (Supplementary Figures S4, S5 respectively). We also assessed associations between 2-hour CEUS score and oxygen consumption; those with signs of improved cortex and medullary perfusion showed increased oxygen consumption by the kidney (Supplementary Figure S6). There was no significant correlation between CEUS metrics and urine flow rate or renal blood flow at the time of the scan, or at 24 h.

### Correlating Human Kidney CEUS Region and Overall Scores With Tissue Apoptosis and NGAL

To generate a single CEUS score for each region, which could be combined to give an overall CEUS score for each kidney, z-score normalised peak and time-to-peak values were calculated. A table of the cohort means and standard deviations which were used to calculate these normalised z-scores, is given in Supplementary Table S4.

**FIGURE 4:**
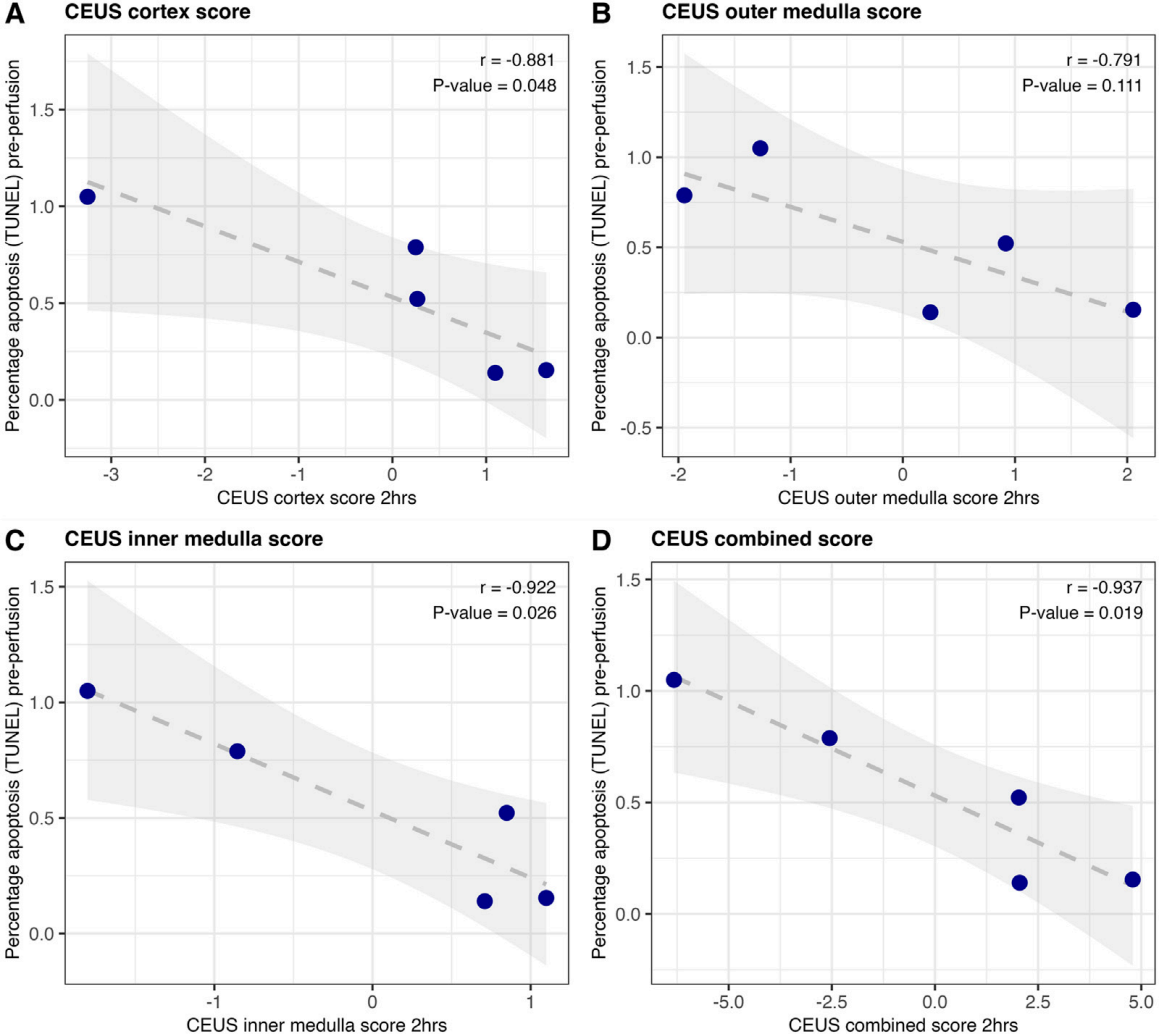
Correlation of region scores with the percentage of cells with DNA fragmentation (a hallmark of apoptosis) on TUNEL at the beginning of human kidney perfusion (n = 5). Region scores for cortex **(A)**, outer medulla **(B)** and inner medulla **(C)** were calculated as peak intensity z-score minus time-to-peak z-score. The three were summed to generate an overall score for the kidney **(D)**. Pearson correlation coefficient and associated p-value are displayed.

As shown in Figures 4A–C these region scores have a negative correlation with tissue DNA fragmentation; kidneys with more DNA fragmentation at the start of perfusion displayed worse perfusion of both cortex and medulla at 2 h. The overall score combining the three regions of interest displayed the strongest correlation with the percentage cells with DNA fragmentation on histology (Figure 4D; Pearson r = - 0.937, P = 0.019).

**FIGURE 5:**
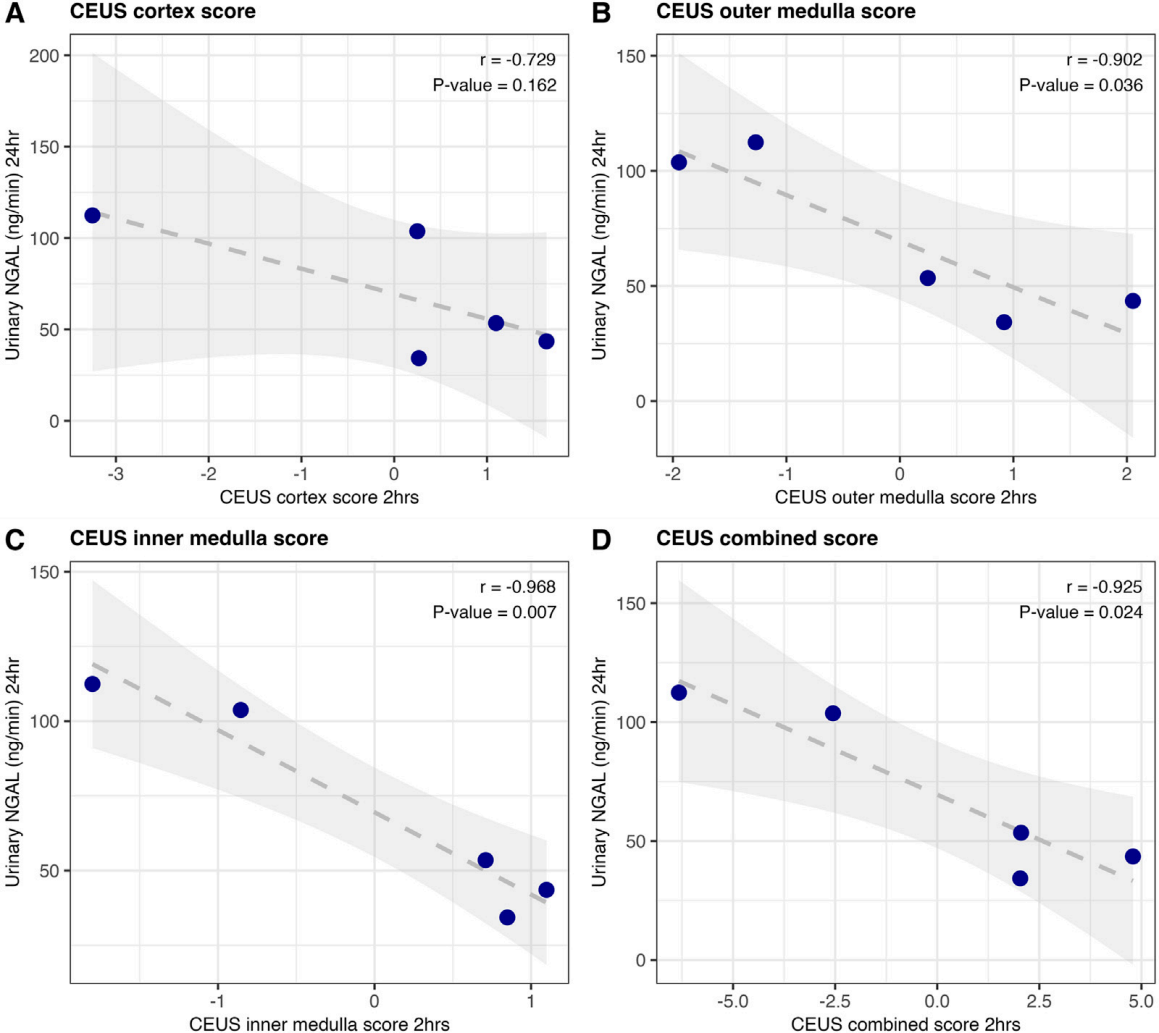
Correlation of CEUS scores at 2 h, with urinary NGAL at 24 h of human NMP (n = 5). Region scores for cortex **(A)**, outer medulla **(B)** and inner medulla **(C)** were calculated as peak intensity z-score minus time-to-peak z-score. The three were summed to generate an overall score for the kidney **(D)**. Pearson correlation coefficient and associated p-value are displayed.

CEUS scores at 2 h were also correlated against 24-h urinary NGAL. Improved CEUS scores at 2 h of NMP were corelated with lower urinary NGAL at the end of perfusion (24 h); Figure 5. This was statistically significant for both outer and inner medulla regions (Pearson r = −0.902, P = 0.036; Pearson r = −0.968, P = 0.007 respectively; Figures 5B, C), and for the overall CEUS score (Pearson r = −0.925, P = 0.024; Figure 5D). Neither CEUS region scores, nor CEUS combined score, showed a significant association with renal blood flow at the time of the scan (Supplementary Figure S7), or the 1-hour “quality assessment score” [2].

### Creation of Web Application and R Package

**FIGURE 6:**
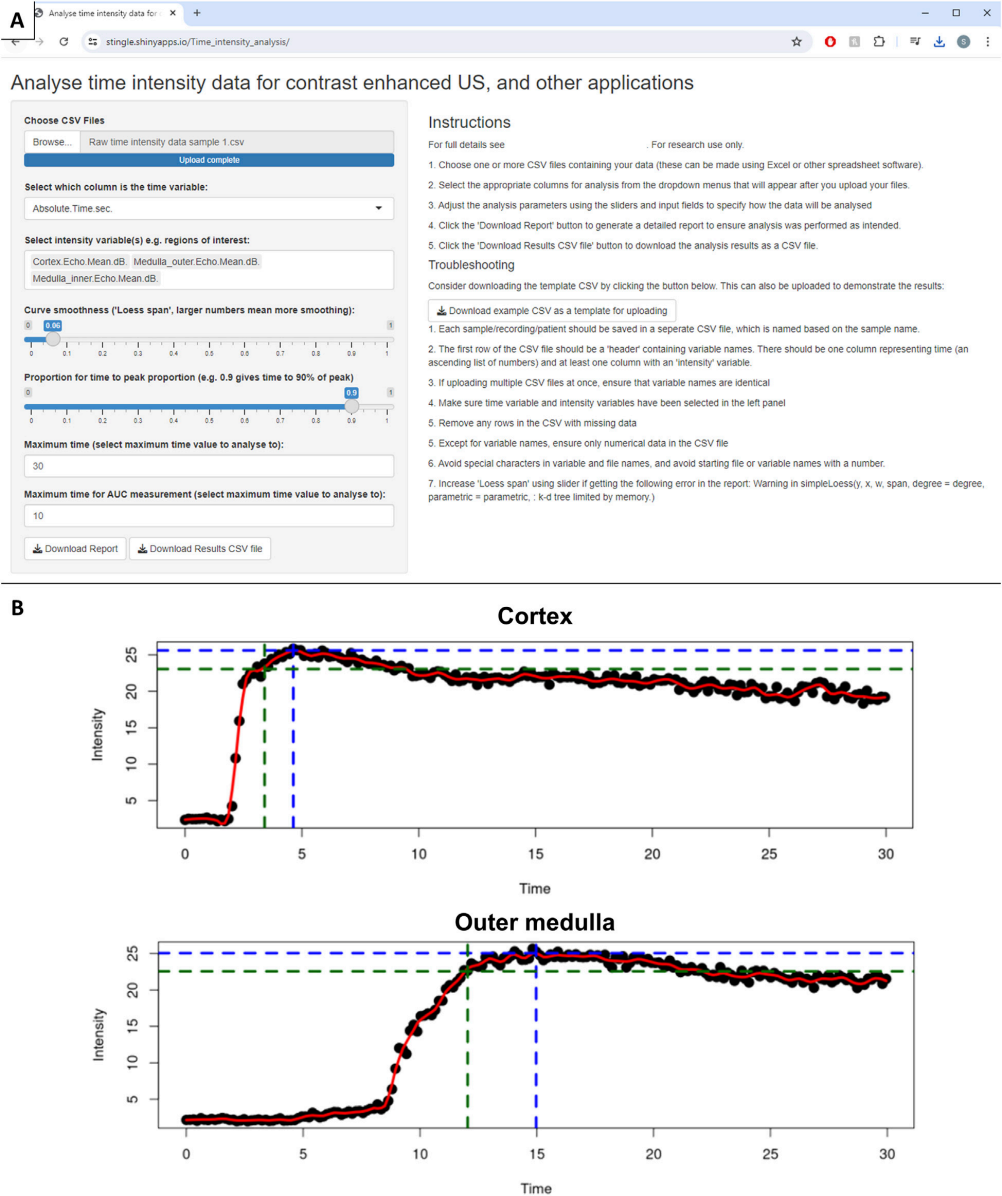
Developed web application for analysis of time-intensity data. **(A)** Web application interface. **(B)** Example intensity time plots from CEUS report. Black dots represent raw data (mean contrast intensity in the given region of interest for that frame). The red line is a LOESS smoother through the raw data. Blue dotted line represents the peak intensity, and time-to-peak intensity, and green dotted line shows 90% of the peak intensity and time until 90% peak intensity.

Following the analyses described above, we wanted to make these techniques accessible to other groups. We have used the shinyapp framework [20] to create a freely available web application [21]. This allows any time-intensity data to be uploaded, and calculates peak and time-to-peak, with additional options for calculating area under the curve and time-to-peak proportion (Figure 6A). A report is also generated to visually confirm the results (Figure 6B). We have also created an R package “tican,” which is freely available to import from CRAN [22], for those who wish to perform these time-intensity curve analyses using R code.

## Discussion

This preclinical study has demonstrated that the ability of contrast-enhanced ultrasound to assess microvascular perfusion can be applied to kidneys during NMP. The quality of microvascular perfusion was associated with tissue DNA fragmentation (TUNEL positive cells as a marker of apoptosis), as well as the damage marker urinary NGAL [17], in both porcine and preclinical human perfusions. Normalisation allows the combination of time-to-peak and peak intensity values into region and overall scores; in human perfusions these scores showed higher correlation with injury markers than any individual metric (Figures 4, 5 versus Supplementary Figures S4, S5). In human kidneys 2-hour CEUS-score was associated with the key biomarker urinary NGAL at 24 h [3, 17]. This is of particular interest as CEUS is a bedside, immediate, non-invasive test with a non-toxic contrast agent [6].

In recent years there has been increasing interest in assessment and reconditioning centres (ARCs) and organ recovery centres (ORCs) to deliver NMP, as a key strategy to assess and recondition marginal organs and increase the donor pool [23–26]. For the ARC concept to be successful, we require validated, real-time assessments of organ quality. These novel techniques are needed both for viability assessment alone, and for assessing potential improvements in organ quality after delivery of advanced therapies. We have previously shown that CEUS could be a powerful tool for assessing response to therapy [9].

Previous work on renal ischaemia reperfusion injury (IRI) provides the biological basis for microvascular perfusion assessment in this setting. Renal IRI is the core pathophysiology damaging organs during retrieval/preservation but is also the core pathophysiology in pre-renal AKI. Previous studies have described intra-renal shunting of blood away from the cortex/medulla to be a hallmark of renal IRI in AKI [4, 5, 27]. A recent study performed microvascular perfusion assessment on patients with AKI versus healthy controls, and found significant decreases in microvascular parenchymal perfusion, despite no change in total renal blood flow [5]. Our data support this; in the porcine setting improved total blood flow is associated with worse microvascular perfusion, which we hypothesise may be due to low resistance shunting. In the human kidneys we found no association between total blood flow and microvascular perfusion; as kidneys were perfused at a fixed pressure this confirms that inferior microvascular perfusion of cortex and medulla seen with CEUS is not related to global or large vessel resistance increases.

Several other viability assessment methods have been developed for use in renal NMP. The best-known is the “quality assessment score” [2, 3]. However, a recent large randomised controlled trial found no association between this score and transplant outcome [28]. Others have suggested potential perfusate or tissue biomarkers which correlate with outcome, such as markers of cell death or inflammation [1, 29]. However, these markers generally cannot be assessed in real time, do not measure function, have shown relatively weak correlation with outcome, and have been selected after screening of multiple potential markers without external validation [29].

This has driven recent interest in utilising imaging modalities to assess organs during NMP [30]. Methods such as MRI and CT have shown some promise, however these would be very challenging to deliver clinically during NMP, as well as introducing risks of nephrotoxic contrast and ionising radiation [30, 31]. In comparison, CEUS is simple to deliver as a point-of-care “bedside” test, gives immediate quantifiable results, and is entirely non-invasive and non-nephrotoxic. It is also relatively easy to perform and there is potential for this to be done by surgeons in the operating theatre prior to transplant. Compared to other modalities CEUS is also relatively inexpensive; whilst costs will vary internationally, in the UK setting the national tariff is less than $100 (USD) per CEUS scan.

To our knowledge this is the first study to focus on CEUS for renal NMP viability assessment, and the first study to assess CEUS for the potential viability assessment of any human organ [30]. Our group has previously applied CEUS during liver NMP in a pilot study, reporting arterial microcirculation improves over the duration of liver NMP [8]. The ability of CEUS to assess the viability of liver, and other perfused organs, is an interesting topic for future research. Novel techniques such as ultrasound localization microscopy offer far higher spatial resolution, which may further delineate microvascular changes during NMP [32]. However, this technique is not in routine clinical practice, as a result of significant disadvantages in terms of device and computing costs [33].

The analysis and quantification of CEUS data, or more broadly any time-intensity data, is relevant outside of the setting of transplantation [5, 31]. Our freely available open source web application (stingle.shinyapps.io/Time_intensity_analysis) and R package (“tican”) could therefore be used in a wide range of research settings, to quantify time-intensity curve data for subsequent analysis [21, 22]. When analysing CEUS recordings, the region of interest assessment could be performed using the ImageJ/Fiji “ROI manager” [34], followed by the use of our tools, to make the entire analysis pipeline free and open-source.

The core limitation of this work is its preclinical nature and the lack of post-transplant data. Clinical validation of this technique is required to translate this preliminary work and assess the ability of CEUS to predict post-transplant outcome. We did not explore trends in CEUS scores over different timepoints in this study; we would be keen to explore CEUS trends in future clinical studies, as these may offer additional information capable of improving our ability to predict post-transplant outcome.

Due to small sample sizes, we focussed on correlation with a relatively small number of NMP outcomes to avoid type 1 error. We attempted to focus on the machine perfusion outcomes which currently have the best evidence for correlating with post-transplant outcome in human; urinary NGAL, urine flow and total blood flow [2, 3, 17]. However, there exists no accurate marker during NMP to act as a “ground-truth” to compare against [28]. Another limitation is the fact that CEUS scores generated here with z-score normalisation are likely specific to the perfusion protocol and imaging system used, and may require adaptation for use in alternative perfusion protocols or ultrasound machines.

In conclusion, CEUS allows point-of-care real-time assessment of microvascular perfusion during renal NMP, which is non-invasive and non-toxic. Techniques for quantification of CEUS were developed and disseminated via open-source software. CEUS-score at 2 h showed correlation with key biomarker urinary NGAL at 24 h of NMP in preclinical human kidneys. This warrants clinical research to assess the ability of CEUS during renal NMP to predict post-transplant outcome.

## Data Availability

The raw data supporting the conclusions of this article will be made available by the authors, without undue reservation.

## Abbreviations

AKI, Acute Kidney Injury; ARC, Assessment and Reconditioning Centre; CEUS, Contrast-Enhanced Ultrasound; CIT, Cold Ischaemic Time; IRI, Ischaemia Reperfusion Injury; MSB, Martius Scarlet Blue; NGAL, Neutrophil Gelatinase-Associated Lipocalin; NMP, Normothermic Machine Perfusion; ORC, Organ Recovery Centre; PI, Peak Intensity; ROI, Region of Interest; TTP, Time-to-Peak; TUNEL, TdT-mediated dUTP Nick-End Labelling; WIT, Warm Ischaemic Time.
