# Supplementary material for: Contrast-Enhanced Ultrasound to Assess Kidney Quality During Ex Situ Normothermic Machine Perfusion
Source: Transpl Int. 2025 Apr 2;38:14268. doi: 10.3389/ti.2025.14268 (PMC11999844; doi:10.3389/ti.2025.14268)
Supplement: Supplementary file 1 [file DataSheet1.PDF]

Supplementary material

Supplementary tables

Supplementary table 1 – porcine 6 hour machine perfusion protocol details.

Perfusion circuit and hardware	• Perfusion circuit (Medtronic – M449802B)<br>• Motor 560A external drive (Medtronic)<br>• Flow waveform: continuous<br>• Heater exchanger (Hico-Variotherm 555, Hico)<br>• Flow probe (TX50P flow transducer, Medtronic)<br>• Bio-console (Bio-console 560, Medtronic)<br>• 3 Syringe drivers (Graseby)
Perfusate components	• 250 mL autologous whole blood containing 10000 I.U. sodium heparin (Panpharma)<br>• 300 mL Aquapharm no.9 Ringers Solution (Animalcare Ltd)<br>• 20 mL 10% mannitol solution (Polyfusor)<br>• 3.75mg dexamethasone (Hameln)<br>• 10 mL 8.4% sodium bicarbonate solution (Freeflex)<br>• 100 I.U. insulin (actrapid)<br>• 40 mg Papaverine Hydrochloride<br>• 0.056g Creatinine (Sigma)
Continuous infusions	• 5% glucose solution at 5 mL/hr (Freeflex)<br>• Epoprostenol (Flolan™ MercuryPharma) at 4 mL/hr<br>• 1 full vial multivitamins (cernevit, Baxter) reconstituted in amino acid solution (Synthamin, Baxter) 17 at 20 mL/hr<br>• Aquapharm no.9 Ringers Solution (Animalcare Ltd) matching fluid loss
Other	• Oxygenated with gas mixture of 95% O2 and 5% CO2 flowing at 100 mL/min.<br>• Ringers infusion was matched to fluid loss to keep reservoir at approximately 500 mL

Supplementary table 2 - Human 24 hour normothermic machine perfusion protocol details

Perfusion circuit and hardware per circuit	• Perfusion circuit (Medtronic – M449802B)<br>• Motor 560A external drive (Medtronic)<br>• Flow waveform: continuous<br>• Heater exchanger (Hico-Variotherm 555, Hico)<br>• Flow probe (TX50P flow transducer, Medtronic)<br>• Bio-console (Bio-console 560, Medtronic)<br>• 3 Syringe drivers (Graseby)
Perfusate components per circuit	• 1 unit (275-320mL) packed red cells in SAG-M.<br>• 100mL of 20% albumin (Alburex® 20, CSL Behring UK Limited)<br>• 170mL Aquapharm no.9 Ringers Solution (Animalcare Ltd)<br>• 20mL 10% mannitol solution (Polyfusor)<br>• 10mL of 10% calcium gluconate<br>• 750mg cefuroxime in 6mL dH_2O<br>• 15mL 8.4% sodium bicarbonate solution in 42mL dH_2O<br>• 5000 units heparin (1000 units/mL) with a further 5000 units added after 12 hours<br>• 10mg Creatinine (Sigma) added once urine recirculation started
Continuous infusions per circuit	• 5% glucose solution mixed with an equal volume of 0.0208 units/mL insulin (Actrapid® diluted in 0.9% sodium chloride) and infused at 8mL/hr<br>• Epoprostenol (Flolan™ MercuryPharma) 1µg/mL in 0.9% saline infused at 4 mL/hr<br>• Amino acid solution (Synthamin 17 without electrolytes (Baxter)) supplemented with 0.5mL reconstituted multivitamin solution (Cernevit, Baxter) per 50mL Synthamin. Infused at 1 mL/hr
Other	• Oxygenated with gas mixture of medical O_2 and medical air flowing at 10 mL/min and 100mL/min respectively.<br>• After an initial period not exceeding 30 minutes in which no more than 10mL of urine was passed, urine was recirculated into the perfusate reservoir to create a closed loop.

Supplementary table 3 – device details and standard operating procedure for contrast enhanced ultrasound

Device details	Philips EPIQ7 Ultrasound machine with QLab 8.1 software (Philips)<br>Probe: eL18-4 probe
Reagent details	Sulphur hexafluoride contrast agent (SonoVue ®, Bracco)
Contrast settings	• Gain for the contrast screen – 60%<br>• C 54<br>• Gen<br>• M10.07
Standard operating procedure	• Activate Philips contrast mode<br>• Contrast side/side<br>• Ensure settings:<br>○ Adjust contrast gain to 60%: this is controlled by the ‘2D’ knob whilst in contrast mode.<br>○ Depth 5cm<br>○ Zoom all the way out with zoom button (not altering depth)<br>○ Ensure image is maximised by toggling MaxVue<br>• Achieve clear view of as many pyramids as possible<br>• Draw up 1mL of reconstituted contrast (SonoVue) into a 5mL syringe<br>• Attach to machine perfusion circuit arterial 3 way tap, and draw up 4mL of perfusate (5mL total volume). Close 3 way tap. Remove any bubbles ready to flush. Ensure no contrast enters the machine perfusion circuit at this stage.<br>• Click on acquire 1 to start the 3 minute loop<br>• Give contrast and click contrast timer on touchscreen<br>• Record for further 65 seconds from contrast entering vessel (use contrast timer on screen as a guide)<br>• Click acquire 1 again to stop recording and save the loop

Supplementary table 4 – human kidney cohort mean and standard deviation for peak and time-to-peak by region. These are the values used to calculate z-scores.

Variable	Mean	Standard deviation
**Peak intensity (dB)**		
Cortex	23.27	1.29
Outer medulla	20.31	1.18
Inner medulla	18.5	2.18
**Time-to-peak (s)**		
Cortex	2.96	2.16
Outer medulla	12.63	2.99
Inner medulla	17.07	5.1

Supplementary figures

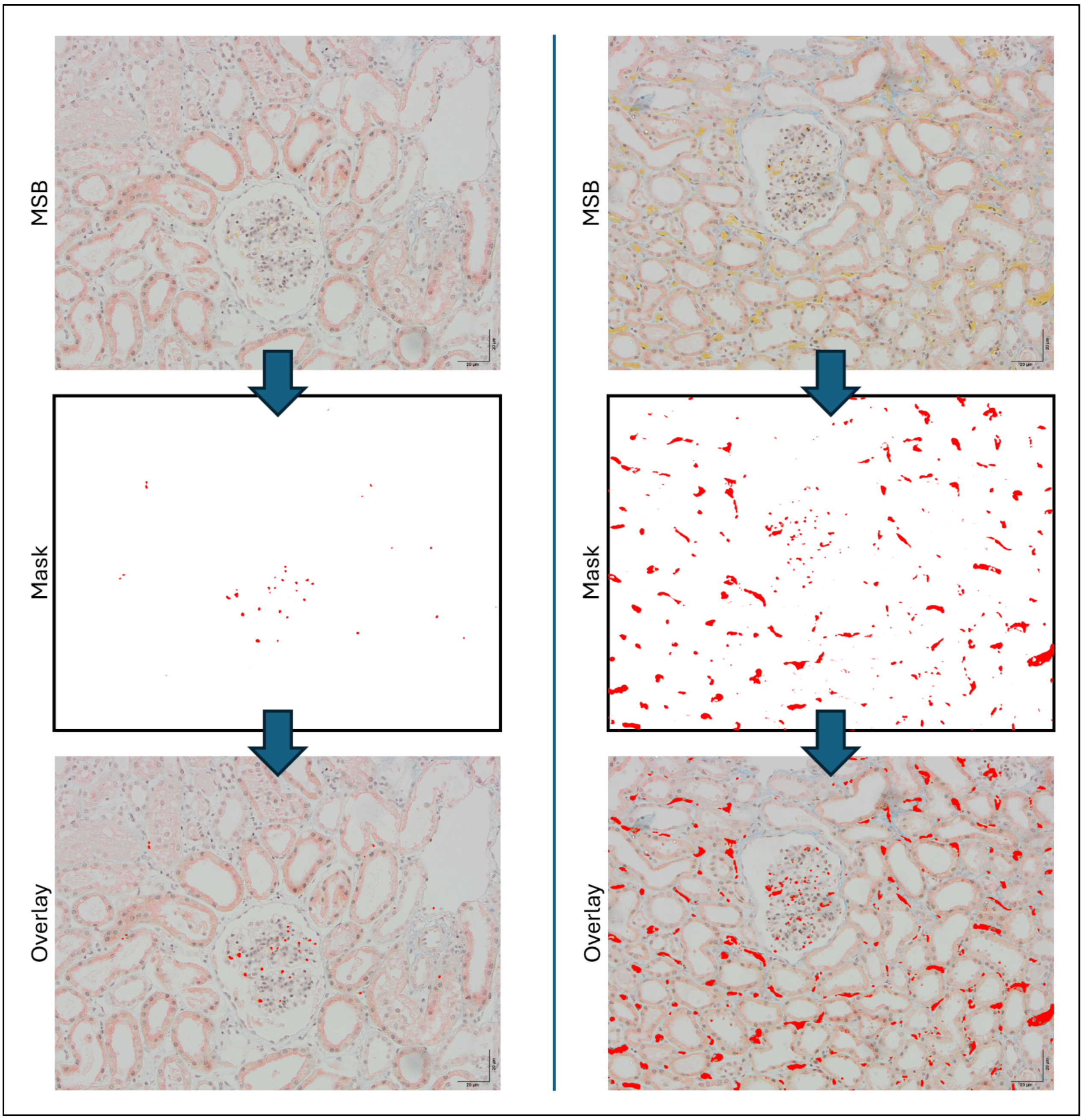

Supplementary Figure 1 – representative Martius scarlet blue stains from 6-hour porcine cortex wedge biopsies (top panels), with fibrin rich red blood cell aggregates (yellow-orange) seen in the microvasculature. Middle panels show generated masks generated by the trained LABKIT classifier. This highlights areas with red blood cell aggregates in red, which are used to calculate the percentage of image pixels representing RBC aggregates. Bottom panels overlay the red areas onto the original image.

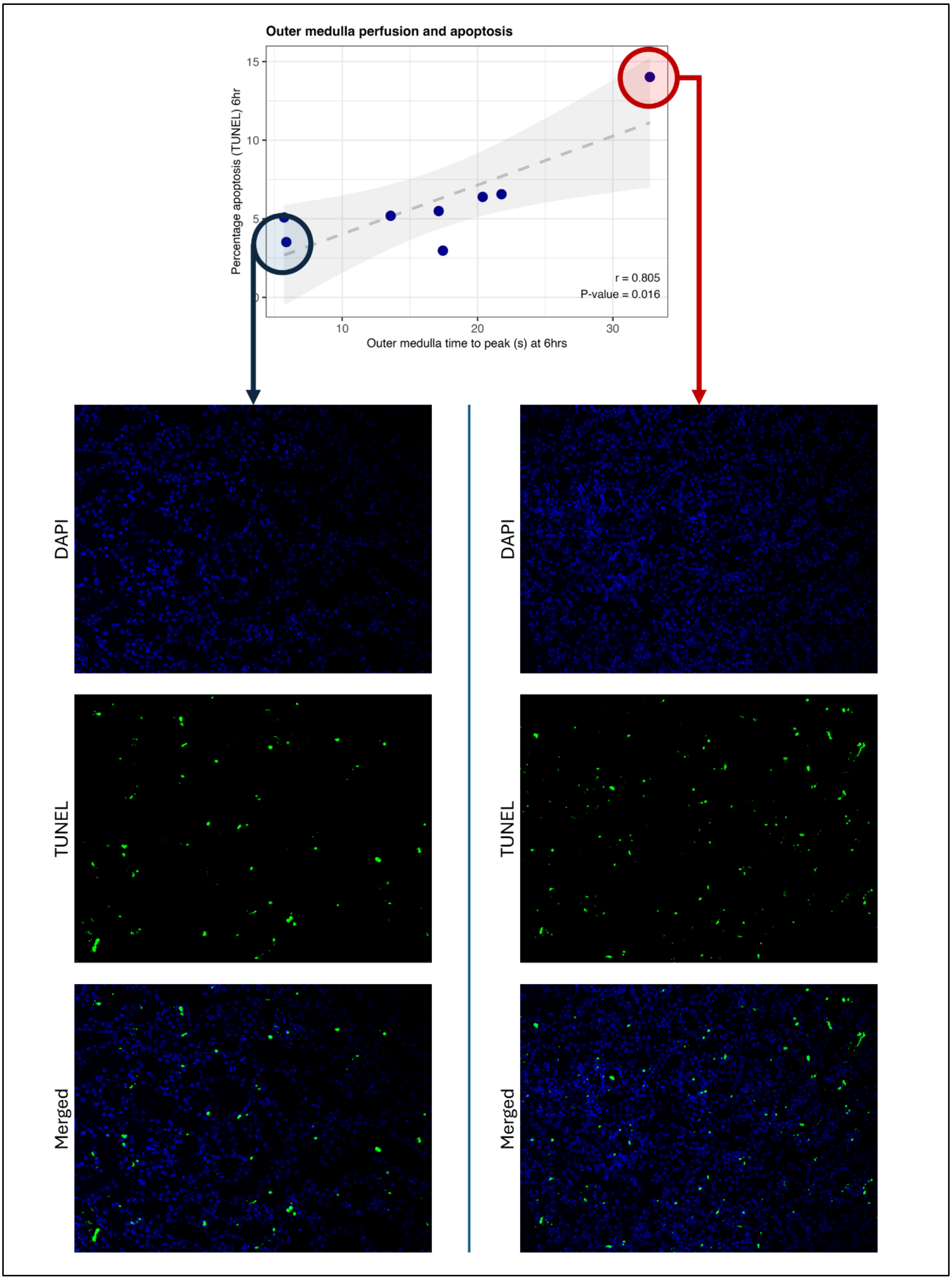

Supplementary Figure 2 - representative terminal deoxynucleotidyl transferase dUTP nick end labeling (TUNEL) images from kidneys with good versus poor microvascular perfusion. Formalin fixed paraffin embedded tissue from 6-hour porcine wedge biopsies. TUNEL positive (representing cell death) is shown in green. Images are counterstained with DAPI (blue) to show nuclei.

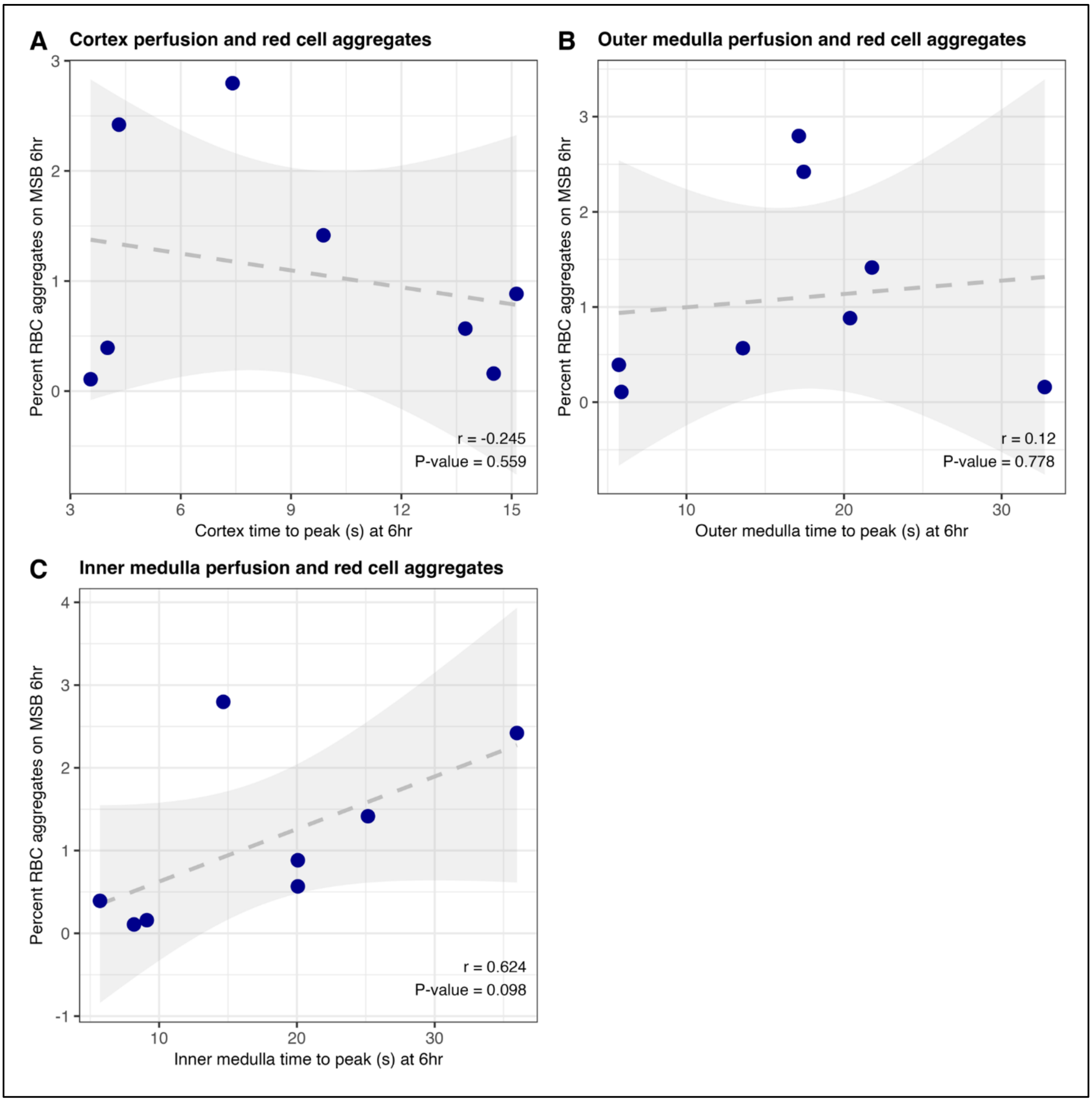

Supplementary Figure 3 - correlation of CEUS results and quantity of red blood cell aggregates in cortex wedge biopsies. Pearson correlation coefficient and associated p-value are displayed. MSB, Martius scarlet blue; RBC, red blood cell.

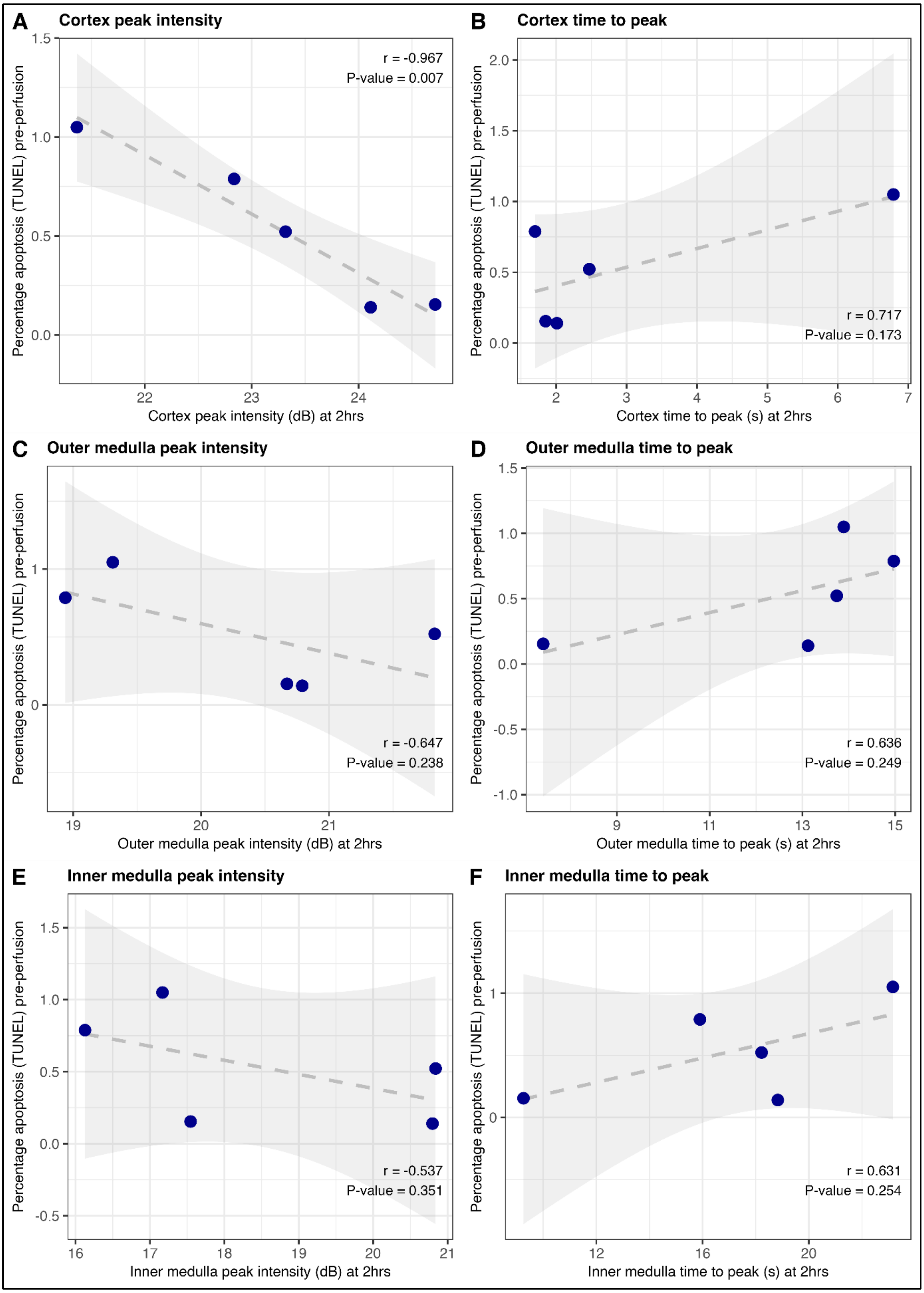

Supplementary Figure 4 - correlation of start of perfusion percentage apoptosis (TUNEL) and 2-hour CEUS microvascular perfusion of cortex (A&B), outer medulla (C&D) and inner medulla (E&F) in human kidneys.

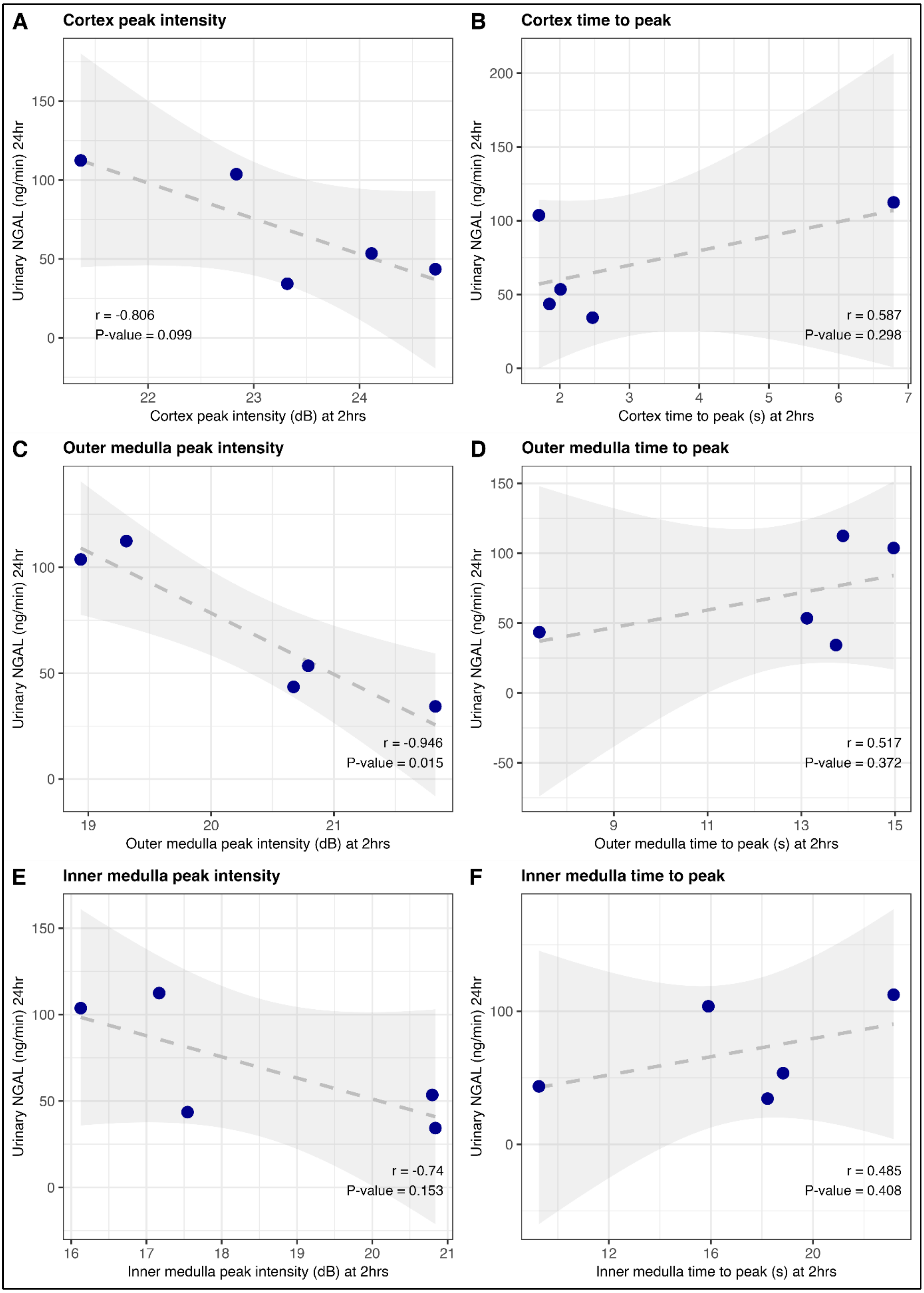

Supplementary Figure 5 - correlation of 2-hour CEUS microvascular perfusion and end of perfusion (24 hour) urinary NGAL in human kidneys. A&B) Cortex. C&D) Outer medulla. E&F) Inner medulla.

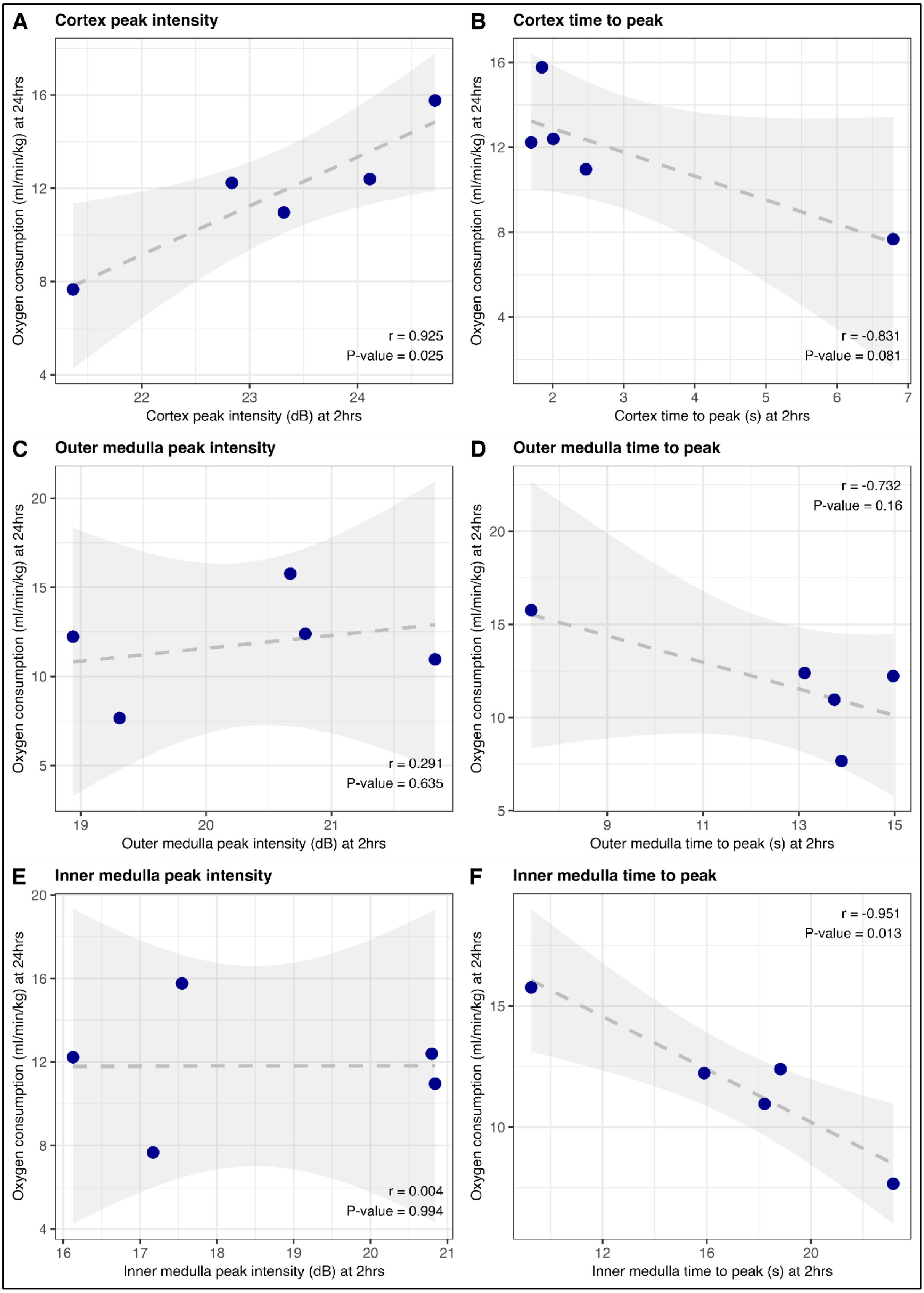

Supplementary Figure 6 - correlation of 2-hour CEUS microvascular perfusion and end of perfusion (24 hour) oxygen consumption in human kidneys. A&B) Cortex. C&D) Outer medulla. E&F) Inner medulla.

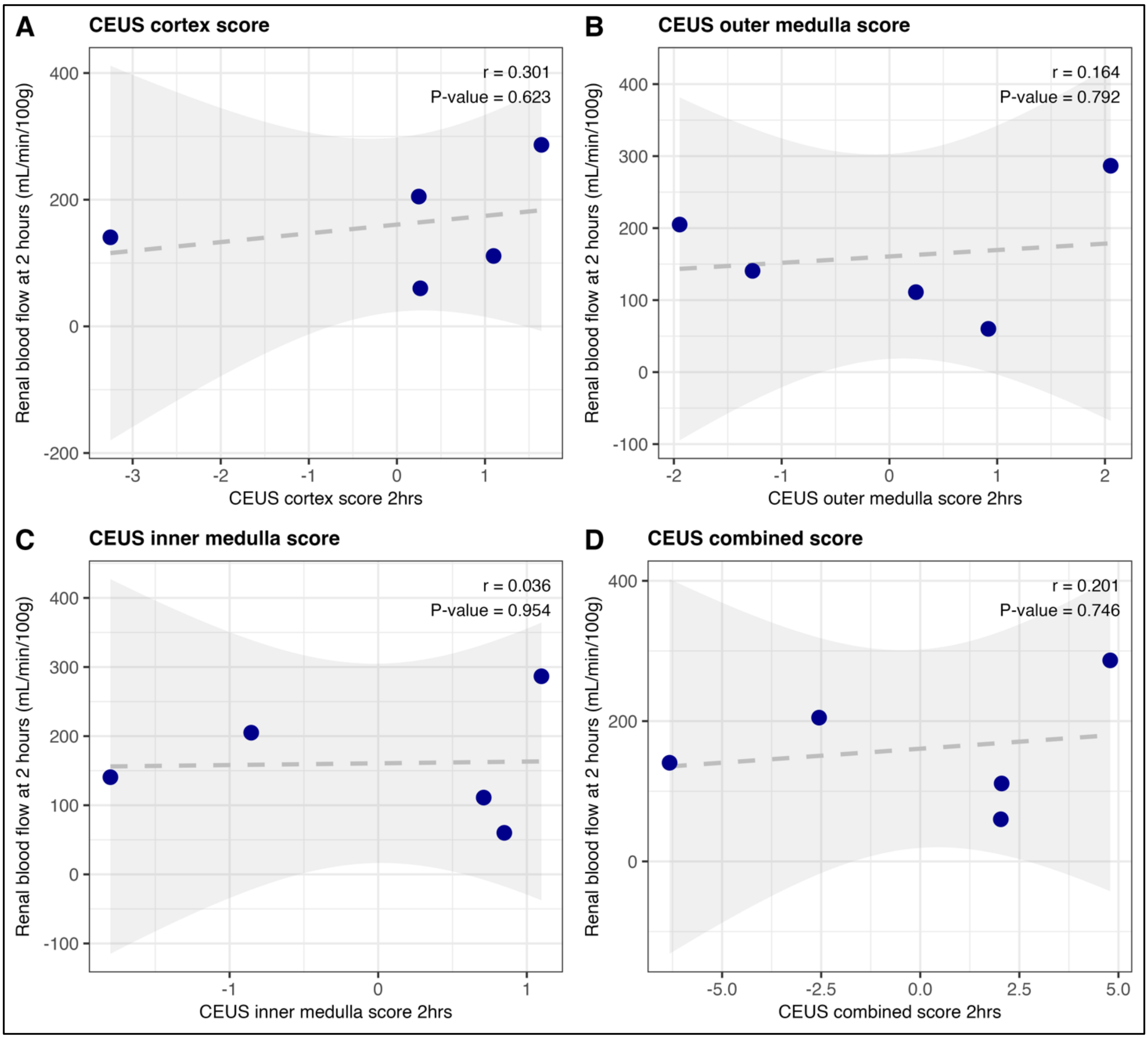

Supplementary Figure 7 - correlation of region scores with total renal blood flow at the time of the scan, in human normothermic machine perfusion. Region scores for cortex (A), outer medulla (B) and inner medulla (C) were calculated as peak intensity z-score minus time-to-peak z-score. The three were summed to generate an overall score for the kidney (D). Pearson correlation coefficient and associated p-value are displayed.
